# Impact of the COVID-19 virus and confinement on the mental health of the tunisian population: Anxiety and depression

**DOI:** 10.1192/j.eurpsy.2021.739

**Published:** 2021-08-13

**Authors:** N. Regaieg, M. Elleuch, S. Hentati, J. Masmoudi

**Affiliations:** 1 Psychiatry “a” Department, Hedi Chaker UHC, Sfax, Tunisia, Sfax, Tunisia; 2 Psychiatry “a” Department, Hedi Chaker University Hospital, Sfax, Tunisia; 3 Psychiatrie “a” Department, Hedi Chaker Hospital University -Sfax - Tunisia, sfax, Tunisia

**Keywords:** COVID-19, Anxiety, Depression, mental health

## Abstract

**Introduction:**

The 2019 Coronavirus disease epidemic is a public health emergency of international concern and poses a challenge to psychological resilience.

**Objectives:**

To study the psychological repercussions in terms of anxiety and depression of the Coronavirus pandemic on the Tunisian population.

**Methods:**

This was a cross-sectional, descriptive and analytical study. We used an online questionnaire on Facebook, on June 2020. The heteroquestionnaire included epidemiological data and two scales: the State-Trait Anxiety Inventory (STAI Form Y-1) to evaluate the anxiety level at the time of the study, and the Patient Health Questionnaire (PHQ 9) to detect a characterized depressive episode.

**Results:**

We included 121 participants. They had an average age of 36.52 years with a sex ratio (M/F) of 0.41. The mean STAI score was 43.12 while the PHQ score was 7.46, indicating that 30.8% of the participants suffered from depression. Both scores were correlated to female sex (p=0.01 for STAI and p=0.02 for PHQ), a history of anxiety (p<0.001) and depressive disorders (p<0.001) and to poor sleep quality (p<0.001). The STAI score was also associated with a family history of high blood pressure (p=0.004), while the PHQ score was correlated to a family history of diabetes (p=0.02), a widowed or divorced marital status (p<0.001) and to a single lifestyle (p=0.03). Furthermore, the two scores (STAI-Y and PHQ 9) were also associated (p<0.001; r=0.67).

**Conclusions:**

The psychological impact of Coronavirus epidemic seems not negligible requiring psychological interventions to improve the mental health of vulnerable groups.

